# Polygenic susceptibility to testicular cancer: implications for personalised health care

**DOI:** 10.1038/bjc.2015.334

**Published:** 2015-10-13

**Authors:** Kevin Litchfield, Jonathan S Mitchell, Janet Shipley, Robert Huddart, Ewa Rajpert-De Meyts, Niels E Skakkebæk, Richard S Houlston, Clare Turnbull

**Affiliations:** 1 Division of Genetics and Epidemiology, The Institute of Cancer Research, London SW3 6JB, UK; 2 Division of Molecular Pathology, The Institute of Cancer Research, London SW3 6JB, UK; 3 Division of Cancer Therapeutics, The Institute of Cancer Research, London SW3 6JB, UK; 4 Academic Radiotherapy Unit, The Institute of Cancer Research, London SW3 6JB, UK; 5 Department of Growth and Reproduction, Copenhagen University Hospital (Rigshospitalet), Copenhagen, Denmark; 6 William Harvey Research Institute, Queen Mary University, London EC1M 6BQ, UK

**Keywords:** testicular cancer, germ cell tumour, TGCT, polygenic risk scoring, disease screening, GWAS

## Abstract

**Background:**

The increasing incidence of testicular germ cell tumour (TGCT) combined with its strong heritable basis suggests that stratified screening for the early detection of TGCT may be clinically useful. We modelled the efficiency of such a personalised screening approach, based on genetic risk profiling in combination with other diagnostic tools.

**Methods:**

We compared the number of cases potentially detectable in the population under a number of screening models. The polygenic risk scoring (PRS) model was assumed to have a log-normal relative risk distribution across the 19 currently known TGCT susceptibility variants. The diagnostic performance of testicular biopsy and non-invasive semen analysis was also assessed, within a simulated combined screening programme.

**Results:**

The area under the curve for the TGCT PRS model was 0.72 with individuals in the top 1% of the PRS having a nine-fold increased TGCT risk compared with the population median. Results from population-screening simulations only achieved a maximal positive predictive value (PPV) of 60%, highlighting broader clinical factors that challenge such strategies, not least the rare nature of TGCT. In terms of future improvements, heritability estimates suggest that a significant number of additional genetic risk factors for TGCT remain to be discovered, identification of which would potentially yield improvement of the PPV to 80–90%.

**Conclusions:**

While personalised screening models may offer enhanced TGCT risk discrimination, presently the case for population-level testing is not compelling. However, future advances, such as more routine generation of whole genome data is likely to alter the landscape. More targeted screening programs may plausibly then offer clinical benefit, particularly given the significant survivorship issues associated with the successful treatment of TGCT.

Testicular germ cell tumour (TGCT) is the most common cancer in men aged 15–45 years (Bray *et al*, 2006; Ruf *et al*, 2014). Germ cell tumours account for over 95% of all testicular cancer, with over 18 000 new cases of TGCT diagnosed annually in Europe (Le Cornet *et al*, 2014). Over the last 30 years there have been significant advances in the treatment of testicular cancer and today, in developed economies, a cure is expected in over 95% of all patients and in around 80% of patients presenting with metastatic disease (Siegel et al, 2012; Oldenburg et al, 2013). The success of treating testicular cancer is however accompanied by long-term consequences associated with survivorship, such as metabolic syndrome, infertility and secondary cancer (Bujan *et al*, 2013; de Haas *et al*, 2013; Rusner *et al*, 2014). Together with the doubling of TGCT incidence over the last 40 years in Western European countries (Le Cornet *et al*, 2014) this has been a strong motivator for raising public awareness about TGCT and consideration of potential screening programs. The benefits of any screening programme can however readily be diminished by excessive false-positive findings and high rates of unnecessary intervention. Importantly, any programme in which screening targets only those who have baseline increased risk will reduce such issues while increasing the detection rates for true disease.

Recognised risk factors for TGCT include a family history of testicular cancer, history of cryptorchidism and past history of germ cell tumour (Trabert *et al*, 2013). Association with various other genitourinary abnormalities has also been variously reported, including microlithiasis, testicular dysgenesis and infertility (Tan *et al*, 2010; McGlynn and Trabert, 2012; Trabert *et al*, 2013). While multiple environmental and *in utero* exposures have been proposed as risk factors, none have been robustly validated (McGlynn and Cook, 2009). Epidemiological studies have consistently demonstrated a 8- to 10-fold increased risk of TGCT in sibling relationships and a 4- to 6-fold increased risk for parent–son relationships (Hemminki and Li, 2004). The strong heritable basis to TGCT risk is supported by twin studies (Swerdlow *et al*, 1997). Migration studies have shown that part of the familial risk is also likely to be influenced by prenatal exposure to as yet unidentified environmental factors (Myrup *et al*, 2008). These studies and the failure of linkage analysis to provide evidence for high-risk TGCT predisposition gene(s) is consistent with much of the inherited familial risk being polygenic, enshrined in the co-inheritance of multiple risk variants, some of which are common. Validation for this proposed model of polygenic predisposition to TGCT has come from recent genome-wide association studies (GWAS), which have so far identified 19 risk loci for TGCT (Table 1) (Kanetsky *et al*, 2009; Rapley *et al*, 2009; Turnbull *et al*, 2010; Kanetsky *et al*, 2011; Turnbull and Rahman, 2011; Chung *et al*, 2013; Ruark *et al*, 2013; Schumacher *et al*, 2013; Litchfield *et al*, 2014). The risk SNPs identified have some of the highest effect sizes reported for any cancer and collectively the 19 risk SNPs explain 15–20% of the excess familial risk of TGCT (Litchfield *et al*, 2015a).

Carcinoma *in situ* (CIS), also termed germ cell neoplasia *in situ* (Ulbright *et al*, 2015), is the non-invasive precursor to TGCT. Molecular and clinical observations are consistent with the first oncogenic transformative step of the progenitor germ cell into CIS occurring during fetal development (Skakkebaek *et al*, 1987; Rajpert-De Meyts, 2006; Kristensen *et al*, 2008). Subsequent proliferation of CIS cells occurs during puberty, likely secondary to hormonal influences (Rajpert-De Meyts *et al*, 2003; Horwich *et al*, 2006). CIS progresses to invasive TGCT within 7 years for 70% of cases and practically 100% will eventually progress (Dieckmann *et al*, 2011). The universal progression of CIS to invasive TGCT is widely accepted and is supported by equivalent rates of CIS/TGCT and by longitudinal studies. CIS is detectable by double-site testicular biopsy in post-pubertal males and therefore provides a reliable pre-invasive biomarker for TGCT. Recently CIS has been also shown to be detectable by immunocytological techniques based on identification of fetal germ cell markers in cells found in semen samples (Hoei-Hansen *et al*, 2007; Almstrup *et al*, 2011).

The genetics of TGCT coupled with CIS acting as a robust biomarker of TGCT offer an attractive schema from which to devise a programme of stratified screening. To explore this possibility we evaluated the predictive discrimination of TGCT-risk SNPs, assessing the application of genetically personalised, multistage population-screening models for TGCT.

## Materials and Methods

### Statistical modelling

The allele frequencies and effect sizes for the 19 TGCT-risk SNPs (Table 1) were obtained from nine published TGCT GWAS (Kanetsky *et al*, 2009, 2011; Rapley *et al*, 2009; Turnbull *et al*, 2010; Turnbull and Rahman, 2011; Chung *et al*, 2013; Ruark *et al*, 2013; Schumacher *et al*, 2013; Litchfield *et al*, 2014), which draw on the following data sets: (i) two independent GWAS data sets, from the United Kingdom (979 cases/4947 controls) and the United States (349 cases/919 controls); (ii) international consortium meta-analysis that combined data sets from point (i) with five other studies to give a total data set of 4242 cases/9566 controls); and (iii) customised follow-up array data (3112 cases/14 026 controls). To examine the predictive value of the 19 known SNPs we constructed polygenic risk scores (PRS) to capture the collective impact of these variants. The 19 variants were assumed to act independently, based on previous statistical testing that showed no evidence of interaction (Ruark *et al*, 2013; Litchfield *et al*, 2014). In brief, the previous tests assessed each pairwise SNP combination using logistic regression, with TGCT as the outcome. The two SNPs were each coded as a categorical variable, while the interaction term (SNP1 × SNP2) were included as continuous covariates; for each pairwise combination of SNPs, the joint risk was consistent with the product of the individual risks. We did not test for evidence of higher-order interactions, that is, between three or more SNPs, however it is unlikely these effects would be reliably detectable, if at all present, using current available methodologies (Wei *et al*, 2014).

The association between TGCT and PRS for all 19 SNPs for an individual is given by:


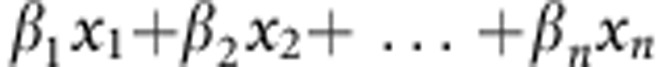


where *β*_*n*_ is the per-allele log OR and *x*_*n*_ the number of risk alleles (either 0, 1 or 2 per locus) carried by each individual at each SNP, and *n* being total number of SNPs. The PRS distribution in the population follows a log-normal distribution LN (*μ*, *σ*^2^) with mean *μ* and variance *σ*^2^ (i.e. relative risk (RR) is normally distributed on a logarithmic scale). As per Pharoah *et al* (2002), in cases the distribution of the PRS is given by: (*μ*+*σ*^2^, *σ*^2^), that is, with same variance *σ*^2^, but with a mean *μ* shifted to the right by *σ*^2^. *μ* and *σ*^2^ are given by:


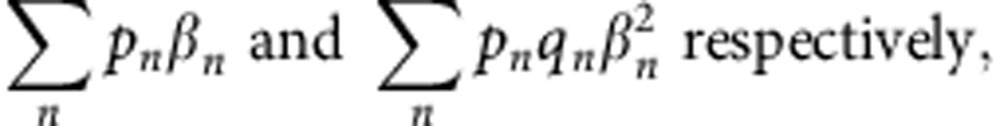


where *p*_*n*_ is the minor allele frequency of the *n*th SNP and *q*_*n*_=1−*p*_*n*_ (Pharoah *et al*, 2002). For the 19 TGCT-risk loci we calculated *σ*^2^=0.74. The mean PRS from the theoretical distribution is an arbitrary value, calculated as exp(*μ*+*σ*^2^/2), and to give a mean RR in the population of 1.0 we set *μ*=−*σ*^2^/2. This model was then used to calculate the predictions of RR for individuals at or above a given percentile. Receiver operator characteristic (ROC) curves were generated using the same case/population distributions as above, across a set of risk thresholds between *μ*±3*σ*^2^. In addition, ROC curve values were also generated for all TGCT predisposition factors, using familial RR estimates from epidemiological studies. Familial RR is assumed to include both genetic and a proportion of environmental risk factors, for example, shared childhood exposures or *in utero* exposures. These values were calculated using:


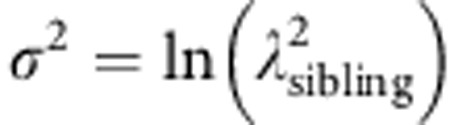


where *λ*_sibling_, was estimated using a familial RR of 8.0 obtained from Hemminki and Li (2004). Population average lifetime TGCT risk was based on 2014 CRUK lifetime incidence rate of 0.5% (CRUK, 2014), multiplied by RR to give lifetime risk per percentile of the PRS. Competing mortality risk analysis was not conducted as over three quarters of TGCT cases present at ages 45 years and younger (CRUK, 2014), for whom cumulative mortality risk from all other causes is only 3.6% (ONS, 2013).

### Modelling population screening

To evaluate the utility of the PRS model for population screening for TCGT, we applied genetic risk data in combination with clinical parameters to a theoretical population of 1 000 000 men. Clinical assumptions and references used in this model are detailed in Table 2 and were taken from four primary sources: Cancer Research UK incidence/mortality statistics, Royal Marsden patient data, a large-scale German study of testicular biopsies by Dieckmann *et al* and semen assay testing completed at Copenhagen University Hospital. Specifically the models were based on a one-off post-pubertal screening of genotype to identify individuals in the top 1% of risk (i.e., 10 000 men), combined with follow-up testing for the top 1% to detect the presence of CIS by semen assay/testicular biopsy. The CIS precursor lesion is assumed to be universally present in all individuals that would go on to develop TGCT. Each testing step (genotyping, semen assay and biopsy) is assumed to have the same diagnostic performance for all men.

## Results

### Utility of PRS for TGCT risk stratification

**Figure 1:**
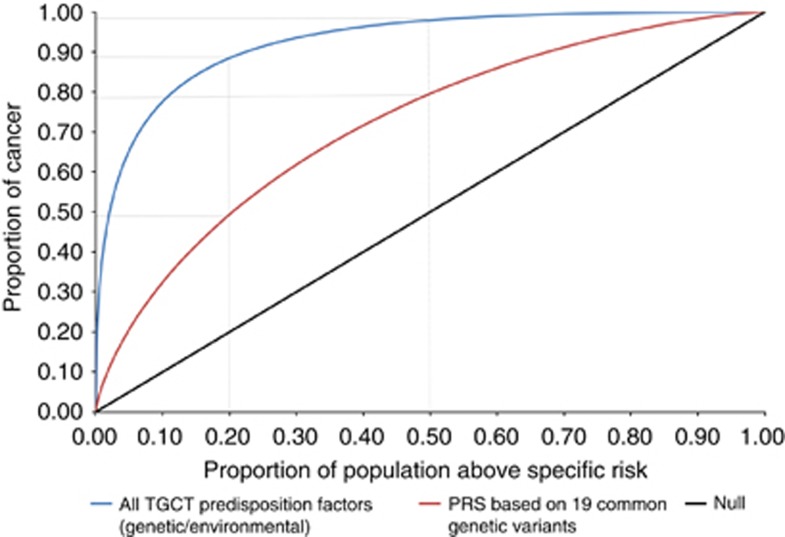
**ROC curve for TGCT predisposition factors.**

**Figure 2:**
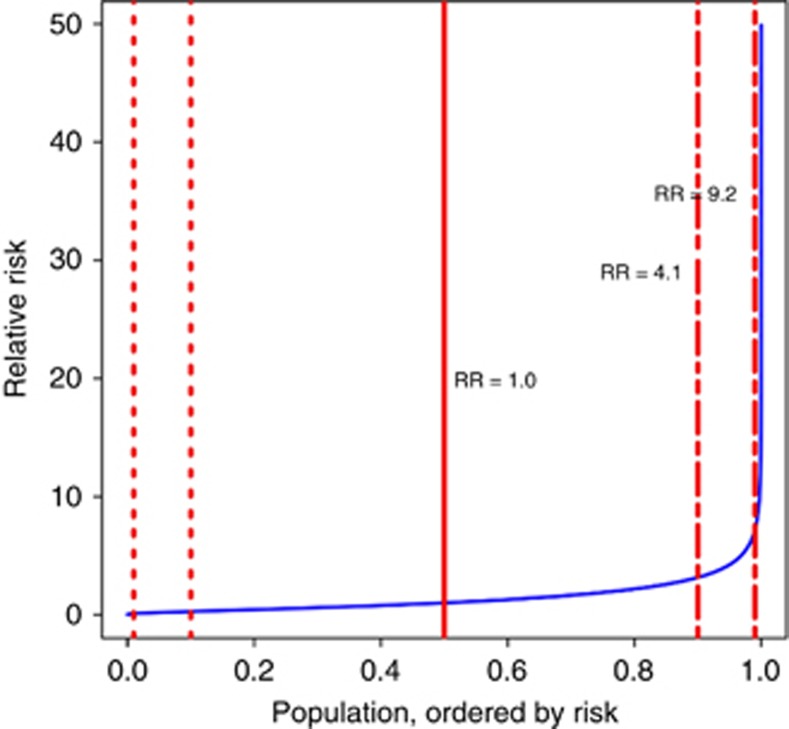
**Population distribution of TGCT relative risk scores ordered by genetic risk (risk is relative to population median risk).** The blue line plots the distribution of RR across the population; the red lines correspond to 1st, 10th, 50th, 90th and 99th centiles. The RR figures presented in black are the average in the (i) highest 10 and (ii) top 1 centile of genetic risk.

Figure 1 shows ROC curves for the PRS model together with the ROC corresponding to all TGCT predisposition factors (genetic and environmental, for reference). The TGCT PRS ROC curve shows that individuals within the top 20% and 50% of genetic risk would account for 48% and 77% of cases, respectively, equivalent figures for all TGCT predisposition factors being 89% and 98%, respectively. The area under the curve for the TGCT PRS model is 0.72. Results from the PRS model shows that men within the top 10% of genetic risk have a 4.1-fold elevated relative risk of TGCT whilst men within the top 1% have a 9.2-fold elevated risk of TGCT compared with the population median (Figure 2).

### Utility of PRS for TGCT screening at a population level

**Figure 3:**
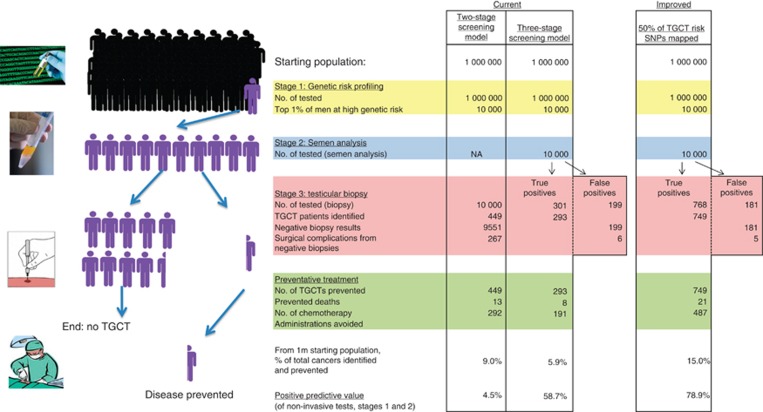
**Simulation of two-stage and three-stage population-based screening for TGCT (see Materials and Methods for references underlying individual clinical assumptions applied).** The improved model (far right) represents a theoretical best case scenario achievable with current technologies. Specific parameters changed in the improved scenario are: TGCT RR for top 1% of men increased to 19.2, equating to an absolute TGCT lifetime risk of 9.6%, an improved semen assay with sensitivity increased from 67 to 80%.

Figure 3 shows the design and outcome of two- and three-stage population-screening scenarios, based on one million men, 0.5% of which would be expected to have CIS and go on to develop invasive TGCT. Under the two-stage screening model, all individuals (one million men) undergo genotype screening across the 19 risk loci, using the PRS model to identify the top 1% of ‘high-risk' individuals (10 000 men). These 10 000 men would then go on to have a bilateral testicular biopsy for detection of CIS. This strategy would lead to the identification of 449 TGCTs (i.e., 10 000 × 0.5% (average lifetime risk) × 9.2 (elevation in risk for top 1%) × 97.5% (biopsy sensitivity)=449); the balancing 9551 biopsies would return a negative result. The 449 identified CIS cases would then be eligible for preventative surgery, which if assumed to completely eliminate TGCT risk, would result in the prevention of 13 deaths and avoidance of chemotherapy in 292 men. Overall two-stage screening will identify only 9.0% of TGCT, with a positive predictive value (PPV) for stage 1 genotyping alone of only 4.5%. The negative predictive value is however high, >99%, reflecting the rarity of TGCT.

We next considered a three-stage screening model (Figure 3), whereby an additional step of semen analysis is conducted after genotype screening but before testicular biopsy. On the basis of published sensitivity of 67% and specificity of 98% (Almstrup *et al*, 2011), this extra step reduces the number of testicular biopsies by 20-fold, whilst still identifying the majority of cases. Under this revised model out of the one million men genotyped, semen analysis is undertaken in 10 000 and the resulting number of biopsies would be 500. This would yield identification of 293 TGCTs, the prevention of 8 deaths and administration of chemotherapy avoided in 191 men. The PPV for non-invasive testing (stages 1+2) increases to 58.7%, however the proportion of population cases identified drops to 5.9%.

### Utility of PRS for TGCT screening at a population level–‘improved' model

To assess the impact of future developments on TGCT-screening viability we modelled an ‘improved' population-screening scenario, assuming that further risk SNPs can be identified and utilised to improve the PRS performance. In order to estimate how many additional SNPs remain still to be discovered we calculated the total potential contribution of common SNPs to TGCT risk using genome-wide complex trait analysis (Yang *et al*, 2011), to calculate the heritability of TGCT. This analysis demonstrated that the heritability of TGCT associated with common genetic variants is 37% (±5.0%) (Litchfield *et al*, 2015b), hence many additional risk SNPs remain to be identified. In the ‘improved' model we added the effect of additional as yet unidentified risk SNPs, assuming that with current sample sizes and genotyping technologies half of all common TGCT SNPs could be realistically identified. Incorporating this additional risk discrimination power into the PRS model resulted in an ‘improved' RR of 19.2 for men in top 1% of risk. Using this ‘improved' PRS model in combination with an ‘improved' semen assay (which we realistically assume has sensitivity improved from 67 to 80%), takes the PPV of the combined test from 59 to 79% (see Figure 3, far right).

## Discussion

The striking effect sizes of TGCT-associated SNPs identified through GWAS, which remain among the highest effect sizes from all GWAS of cancer, have repeatedly raised enquiry as to whether TGCT would be the paradigm through which common genetic variation could first be used clinically to stratify risk (Chanock, 2009). Indeed, the TGCT SNPs demonstrate strength in terms of risk discrimination, for example, the top 1% of highest risk genotypes had a 9.2-fold elevation in TGCT risk, significantly greater than comparable PRS models of ovarian, breast and prostate cancers with comparative risk figures of 1.9, 3.2 and 4.7, respectively (Bahcall, 2013). This is particularly noteworthy when considering that the PRS model for TGCT is based on the inclusion of only 19 loci, compared with the 71 for breast cancer and 77 for prostate cancer included in their respective models (Eeles *et al*, 2013; Michailidou *et al*, 2013). This performance reflects the high effect sizes of the TGCT SNPs. The value of the TGCT PRS must however be considered in a broader clinical context and a number of issues need to be considered alongside the PRS. First, as TGCT is rare with a lifetime male Caucasian absolute risk of 1 in 200, the high relative risks only translate to modest absolute risks. For example, the top 1% of men with a RR of 9.2 have only a 4.6% lifetime risk. Second, the high cure rate for TGCT limits the impact of screening and early detection of disease on disease mortality. Third, the invasive and costly nature of testicular biopsy, which with a reported surgical complication rate of around 3% (Dieckmann *et al*, 2011), renders it challenging to justify applying this procedure to any large population group. However, advances in non-invasive semen assays to detect CIS, particularly if they can be scaled to high-throughput tests with improved sensitivity, offer real opportunity for transformation of screening for TGCT and avoidance of unnecessary biopsy. Indeed with screening based on a three-stage model the number of required biopsies is reduced significantly by the addition of semen analysis into the protocol. Overall the combination of genetic risk profiling and semen assay likely offers greater clinical utility than genetic risk profiling alone. For example, even using the combination of current genetic PRS (with just 19 SNPs) and current semen assays (with sensitivity of 67%) has the potential to achieve a PPV of nearly 60% when testing an unenriched general population. This is a significant increase from the starting TGCT prevalence of ∼0.5% overall. Further research progress is expected in the fields of both genetic predisposition and semen assays. To explore how these developments might affect TGCT screening we constructed an ‘improved' screening model, to represent a theoretical best case scenario achievable with current technologies. This analysis showed men in the top 1% of risk would have a nearly 20-fold increase in TGCT risk, and the PPV of a combined test increases to ∼80%. As a yardstick of total potential, full mapping of all common TGCT SNPs, as estimated from our genome-wide complex trait analysis, would yield a PPV >90%, with power to detect and prevent nearly 50% of TGCTs.

An additional screening approach could be simultaneous testing, with both genetic risk profiling and semen assay testing conducted together in one stage. This approach would have some challenges, for example, given the high specificity (98%) of the semen assays a positive result on this test would over-ride any result from genetic testing. However, in the scenario of a negative semen assay, due to the low sensitivity of the test (67%), then genetic testing could be informative in determining whether the semen assay is a true negative or false negative. Overall cost considerations would likely preclude population-wide semen assay testing, and given the rare nature of TGCT, a risk prioritisation stage is likely to be required to identify high-risk men for whom semen testing/biopsy is cost-effective.

A limitation of the current study is the exclusion of non-genetic risk factors, such as cryptorchidism, from the risk scoring model. These factors are likely to offer additional discriminatory power, however explicit delineation of the interaction between genetic and non-genetic factors has yet to be established. Hence, complex modelling trained and tested on clinical data sets fully characterised for genotype and phenotype, would be of significant interest. Indeed if independence of effect can be proved, a model of targeted screening in men with already elevated TGCT risk as evident from their family or medical history may represent a more immediately tractable model for clinical utility. An additional limitation is that health economic analysis has not been considered in our modelling; however, any large-scale programme is currently unlikely to demonstrate savings in health-related expenditure, given the required genotyping of a large initial population. This paradigm is unlikely to shift unless population-level genomic testing becomes routine practice (for example, by way of delivering neonatal screening, although this has ethical implications (Kerruish and Robertson, 2005)), in which context the disease-associating markers could then be routinely queried from broader genomic databases, without incurring additional screening infrastructure or genotyping costs.

Overall the low absolute disease risk and the marked effectiveness of existing treatment for TGCT mean current data do not support a clear compelling case for programs of population-level genetic screening for TGCT. However, analysis of future developments suggests a longer-term clinical benefit may exist. Furthermore, there may be more immediate potential benefits from TGCT-screening programs targeting those at elevated *a priori* risk. The motivation for further developing these risk models for TGCT is clear, in terms of reducing the occurrence of invasive cancer arising in young men, reducing the burden of chemotherapy-related survivorship issues and reducing mortality in the minority with treatment-refractory disease state.

## Figures and Tables

**Table 1:** TGCT predisposition loci used as input for polygenic risk scoring model

**SNP**^a^	**Gene**	**Band**	**Reference(s)**	**Risk allele frequency**	**Per-allele OR**
rs995030/rs1508595^b^	*KITLG*	12q21	Kanetsky *et al*, 2009; Rapley *et al*, 2009	0.80/0.83	2.55/2.69
rs210138	*BAK1*	6p21	Kanetsky *et al*, 2009; Rapley *et al*, 2009	0.20	1.50
rs4624820	*SPRY4*	5q31	Rapley *et al*, 2009	0.54	1.37
rs4635969	*TERT/CLPTM1L*	5p15	Turnbull *et al*, 2010	0.20	1.54
rs755383	*DMRT1*	9p24	Turnbull *et al*, 2010	0.62	1.37
rs2900333	*ATF7IP*	12p13	Turnbull *et al*, 2010; Kanetsky *et al*, 2011	0.62	1.27
rs8046148	*HEATR3*	16q12.1	Ruark *et al*, 2013	0.79	1.32
rs2839243	Non-coding	21q22.3	Ruark *et al*, 2013	0.47	1.26
rs3805663	*CATSPER3/PITX1*	5q31.1	Ruark *et al*, 2013	0.63	1.25
rs10510452	*DAZL*	3p24.3	Ruark *et al*, 2013	0.70	1.24
rs2720460	*CENPE*	4q24	Ruark *et al*, 2013	0.62	1.24
rs7010162	*PRDM14*	8q13.3	Ruark *et al*, 2013	0.62	1.22
rs9905704	*RAD51C/TEX14/PPM1E*	17q22	Chung *et al*, 2013	0.68	1.21
rs3790672	Non-coding	1q24.1	Ruark *et al*, 2013; Schumacher *et al*, 2013	0.28	1.20
rs2072499	Non-coding	1q22	Chung *et al*, 2013; Ruark *et al*, 2013	0.35	1.19
rs4888262^c^	*RFWD3*	16q22.3	Chung *et al*, 2013	0.458	1.21
rs12699477	*MAD1L1*	7p22.3	Chung *et al*, 2013	0.38	1.16
rs17021463	*HPGDS*	4q22.2	Chung *et al*, 2013	0.42	1.15
rs1510272	*SSR3/TIPARP*	3q25	Litchfield *et al*, 2014	0.73	1.16

Abbreviations: OR=odds ratio; SNP=single-nucleotide polymorphism; TGCT=testicular germ cell tumour.

a For loci with multiple reported SNPs the marker listed is taken from first study referenced in column four.

b Locus 12q21 has two SNPs reported with independent effect (*P*=0.0006, (Rapley *et al*, 2009)), however only rs995030 is included in our polygenic risk scoring model.

c At 16q22.3 data for published SNP rs4888262 were not available in our data set, proximal SNP rs4888265 (which lies in the same linkage disequilibrium block (*R*^2^=1.0)) as the published SNP was used instead. Both these SNPs have comparable OR effect sizes and disease-associating *P*-values in our data sets.

**Table 2:** Clinical assumptions used for population-screening example

**Assumption**	**Value (%)**	**Reference**
Lifetime risk of TGCT	0.5	CRUK, 2014
TGCT mortality rate	2.8	September 2014
Frequency of surgical complications from testicular biopsy	2.8	Dieckmann *et al*, 2011
Semen assay – sensitivity	67.0	Almstrup *et al*, 2011
Semen assay – specificity	98.0	Almstrup *et al*, 2011
Overall rate of chemotherapy administration in TGCT	65.0	Estimate from Royal Marsden Hospital patient data
Genotyping uptake in population	100.0	Theoretical estimate
Sensitivity of testicular biopsy to detect CIS	97.5	Dieckmann *et al*, 2011
Remaining risk of progression to invasive TGCT, following CIS detection and preventative orchidectomy	0.0	Theoretical assumption

Abbreviations: CIS=carcinoma *in situ*; TGCT=testicular germ cell tumour.
