# Polygenic susceptibility to testicular cancer: implications for personalised health care

**DOI:** 10.1038/bjc.2016.136

**Published:** 2016-05-26

**Authors:** Kevin Litchfield, Jonathan S Mitchell, Janet Shipley, Robert Huddart, Ewa Rajpert-De Meyts, Niels E Skakkebæk, Richard S Houlston, Clare Turnbull

**Correction to:** *British Journal of Cancer* (2015) **113**, 1512–1518; doi:10.1038/bjc.2015.334; published online 13 October 2015

**Updated online 26 May 2016:** This article was originally published under a CC BY-NC-SA 4.0 license, but has now been made available under a CC BY 4.0 license. The PDF and HTML versions of the paper have been modified accordingly.

